# Retroperitoneal laparoscopic adrenalectomy for large adrenal tumors—analysis of tumor size and adverse events: a retrospective single-center study

**DOI:** 10.3389/fsurg.2023.1284093

**Published:** 2024-01-05

**Authors:** I-Chen Tsai, Yu-Che Hsieh, Wen-Hsin Tseng, Chien-Liang Liu, Chung-Han Ho, Chien-Feng Li, Allen W. Chiu, Steven K. Huang

**Affiliations:** ^1^ Division of Urology, Department of Surgery, Chi Mei Medical Center, Tainan, Taiwan; ^2^ The Doctoral Program of Clinical and Experimental Medicine, National Sun Yat-Sen University, Kaohsiung, Taiwan; ^3^ Division of Uro-Oncology, Department of Surgery, Chi Mei Medical Center, Tainan, Taiwan; ^4^ Department of Medical Research, Chi Mei Medical Center, Tainan, Taiwan; ^5^ Department of Information Management, Southern Taiwan University of Science and Technology, Tainan, Taiwan; ^6^ Department of Pathology, Chi Mei Medical Center, Tainan, Taiwan; ^7^ Department of Urology, Mackay Memorial Hospital, Taipei, Taiwan

**Keywords:** adrenal tumor, adverse events, outcomes, postoperative complications, retroperitoneal laparoscopic adrenalectomy, tumor size

## Abstract

**Introduction:**

Adrenal tumors are relatively common, and adrenalectomy is the third most common endocrine surgery. Patients with adrenal tumors were categorized into two groups for analysis: those with intermediate (4–6 cm, Group 1) and large (>6 cm, Group 2) tumors undergoing Retroperitoneal Laparoscopic Adrenalectomy (RLA). The primary outcome is to compare the surgical outcomes between these two groups. The secondary outcome involves analyzing the relationship between tumor characteristics and the incidence of adverse events.

**Methods:**

Data from 76 patients who underwent RLA for tumors of size ≥4 cm between 2005 and 2022 at a single tertiary referral center were analyzed retrospectively. Variables, including patients' age, hormone function, operation time, conversion to open approach, perioperative complications, and adverse surgical events (blood loss >500 cc, conversion to open approach, and perioperative complications), were assessed.

**Results:**

No significant differences were observed between the two groups in terms of functional and histopathologic analysis, gender distribution, functioning factors, perioperative complications, and estimated blood loss. However, patients in Group 2 were younger (median age 50, IQR: 40–57, *P* = 0.04), experienced longer operative times (median 175 min, IQR: 145–230 min, *P* = 0.005), and had a higher rate of conversion to open surgery (12%, *P* = 0.033). For every 1 cm increase in tumor size, the odds ratio for adverse surgical events increased by 1.58.

**Conclusions:**

RLA is a safe and feasible procedure for adrenal tumors larger than 6 cm. While intraoperative and postoperative complications are not significantly increased in either group, larger tumors increase surgery times and are more likely to require conversion to open surgery. Therefore, caution and preparedness for potential adverse events are recommended when dealing with larger tumors. A tumor size of 5.3 cm may serve as a guide for risk stratification and surgical planning in large adrenal tumor management.

## Introduction

Adrenal tumors are relatively common, and adrenalectomy is the third most common endocrine surgery (1). The size of large adrenal tumors (LATs) is with a consensus around 6 cm (2, 3). LATs are relatively rare, accounting for 8.6%–38.6% of all adrenal tumors. LATs are often associated with malignancies. The incidence of malignant LATs is approximately 25% (3, 4).

Laparoscopic adrenalectomy has emerged as the standard surgical procedure for adrenal tumors, given its advantages of reduced postoperative pain, shorter hospital stay, and quicker recovery compared to traditional open surgery (5). Nevertheless, the management of LATs (>6 cm) remains challenging and a debated issue because of potential difficulties associated with their resection, and concerns about malignancy. Laparoscopic adrenalectomy for adrenal tumors, including transperitoneal and retroperitoneal approaches, is considered the gold standard for management. The tumor diameter is an essential factor for surgical management of LATs via the retroperitoneal approach.

Retroperitoneal laparoscopic adrenalectomy (RLA) has been reported to have satisfactory clinical results for small adrenal tumors (6–8). This approach, although technically demanding, offers the potential for a direct and shorter route to the adrenal glands, avoidance of intraperitoneal organs, and a decreased risk of contamination in cases of malignancy (9, 10). A previous report has shown that the largest benign adrenal tumor managed by the retroperitoneal approach was 6 cm (6, 7, 11). However, RLA for LATs has still been widely accepted. A recent report showed that both RLA and transperitoneal laparoscopic adrenalectomy (TLA) provide similar results for LATs. RTA showed a greater advantage for management of benign LATs to reduce hormone supplementation, but the number of cases is relatively small (12).

The present report lacks large-scale research to determine whether the use of RLA is appropriate for LATs. We specifically analyzed the adrenal tumors of ≧4 cm and divided them into intermediate and large sized groups. The primary outcome of this study is to comprehensively analyze the results of LATs treated with RLA, and compare the perioperative complications and outcomes between intermediate tumors (4–6 cm) and large tumors (>6 cm), to determine the possible limitations of RLA treatment of LATs. The secondary outcome is to analyze the relationship between tumor size and adverse surgical events to understand how tumor size impacts surgery outcomes, and to determine the appropriate tumor size for risk stratification.

## Methods

### Study design and sample

In this retrospective study, patients with adrenal tumors who were referred to the Department of Urology for a complete adrenal tumor work-up and surgical management (open, RLA and TLA) between January 2005 and October 2022 were screened initially. The inclusion criteria for this study were: (1) Patients were diagnosed as having an adrenal tumor by image survey (Computed Tomography or Magnetic Resonance Imaging) and had received retroperitoneal laparoscopic adrenalectomy (RLA). (2) Baseline tumor indicators and perioperative parameters were fully recorded; (3) The tumor diameter was larger than or equal to 4 cm. The exclusion criteria were: (1) Insufficient data were reported; (2) The tumor was operated via open approach or transperitoneal approach; (3) Bilateral disease; (4) Peripheral invasion was noted on image survey. We analyzed the records of abnormal hormone secretion from adrenal tumors, including serum cortisol, aldosterone, renin and urine vanillyl mandelic acid, nor-adrenaline, adrenaline, dopamine.

All patients provided signed informed consent to receive the operation after we elaborated the benefits and potential risks of the surgery. We also informed all patients about the possibility of conversion to open surgery, which might happen if any difficulties were encountered during the surgery.

Patients who received RLA were divided into those with tumor size 4–6 cm and >6 cm for analysis. Data on patients' age, sex, hormone function, tumor side, operation time, postoperative complications, conversion to open procedure, and rate of malignancy were analyzed.

In this study, adverse events were defined as blood loss greater than 500 ml, conversion to open surgery, intra-operative complications (hemodynamic instability during operation, tumor capsule rupture, massive bleeding and peripheral organ injury) and postoperative complications (Clavien-Dindo Classification grade II or above). Since larger tumors are more likely to pose risks during surgery, related risk factors, such as tumor size and tumor characteristics, were investigated. Factors associated with adverse events based on the size of the tumor were also evaluated.

### Surgical technique

All patients received the retroperitoneal approach. Patients were placed in the lateral decubitus position. The retroperitoneal space was created by balloon dilator. A 30-degree laparoscope was applied as an observation mirror through a 12-mm balloon trocar (Kii ® balloon blunt tip trocar made by Applied Medical, CA, US), and the other two trocars, with a diameter of 5 mm and 12 mm respectively, were placed at the anterior axillary line and posterior axillary line of the subcostal space. The steps of the operation were performed sequentially, as follows: (1) Identify the edge of the adrenal gland; (2) Ligate the central adrenal vein by hem-o-lok® clips; (3) After the adrenal gland was fully dissected, a specimen retrieval bag was used to dress the adrenal gland via a 12-mm trocar. Finally, the tumor was transected and examined on the operating table to ensure resection of the entire tumor. After the space of the adrenal area was exposed, the tumor location was carefully evaluated and we determined whether there was peri-adrenal involvement. Our principle for malignant adrenal tumors is to resect as much periadrenal tissue as possible to ensure the negative margin rate.

A drainage tube was placed routinely after all procedure was done. Our cases were all performed by three experienced senior laparoscopic surgeons (Dr. Huang, Dr. Liu and Dr. Tseng) to reduce the selective bias.

### Postoperative management

After the operation, the patients were routinely transferred to the post-anesthesia care unit for two hours. After the vital signs were stable and consciousness was clear, the patients were sent to the urology ward. Postoperative intravenous fluid supplementation, metoclopramide for bowel movements, hormone supplement (hydrocortisone for one day then shift to prednisolone), pantoprazole for ulcer prevention were prescribed for all patients. Monitoring vital signs and lab data was continued. Drainage tube contents were observed, and if the drainage volume was less than 50 ml, the drainage tube was extubated. If postoperative recovery was good, the patients were discharged from the hospital 2–5 days after the surgery.

### Definitions

In compliance with the 2017 WHO classification of endocrine tumors, all pheochromocytomas are considered potentially metastatic, thus categorized as malignant tumors (11). The intra-operative factors taken into account are the estimated blood loss (EBL), operation time, intra-operative complications, conversion to open procedure, and ICU admissions.

Intra-operative complications included hemodynamic instability during operation, tumor capsule rupture, massive bleeding and peripheral organ injury.

Hemodynamic instability refers to the situation where blood pressure can either spike to 200 to 300 mmHg during tumor manipulation or suddenly drop after the tumor is removed, necessitating the use of inotropes.

Massive bleeding refers to intraoperative bleeding of more than 500 cc or the need for a blood transfusion during surgery.

Postoperative outcomes involve length of hospital stay, postoperative complications and the recurrence of malignant disease. Postoperative complications pertain to complications (Clavien-Dindo Classification grade II or above) that occur during the hospitalization period or within 30 days postoperatively. Postoperative complications included postoperative pulmonary edema requiring diuretic treatment, adrenal insufficiency, and low blood pressure necessitating inotropic treatment.

### Statistical analysis

Categorical variables are reported as frequencies with percentages and evaluated using Pearson's chi-square tests or Fisher's exact test to compare the differences between the two groups. The normal distribution of continuous variables, namely age, BMI, operative time, hospital stay, blood loss, and tumor size, was assessed using the Kolmogorov-Smirnov or Shapiro-Wilk normality test. Since all *P*-values were <0.05, it was determined that these variables did not meet the normality assumption. As the continuous variables did not pass the normality test, they are presented as medians with interquartile ranges (IQR) and compared using the Wilcoxon rank sum test.

To determine the appropriate cut-off point, the Youden index approach was used according to the area under the ROC curve. Logistic regression was used to estimate associations between the variables of interest and adverse factors by calculating the odds ratios (ORs) and 95% confidence intervals (95% CIs). All significant variables were adjusted for the multivariable model to correct for possible confounders. All statistical analyses were performed using SAS software, version 9.4 (SAS Institute Inc., Cary, NC, USA), and the significance level was set at 0.05 for all two-tailed statistical tests.

## Results

From January 2005 to October 2022, 442 patients with adrenal tumors were analyzed; 366 were excluded, and 76 met our inclusion criteria. Of these, we divided them into two groups based on tumor size: Group 1 consisted of 51 patients with tumors measuring between 4 and 6 cm, while Group 2 included 25 patients whose tumors were larger than 6 cm, with sizes ranging from 6.5 to 11.5 cm. The average size of the tumors in Group 2 was 7.896 cm, with the median being 7.7 cm. The study consisted of 34 (44.7%) male and 42 (55.3%) female patients with an age range of 17–80 years (median age: 55 years) (Table 1).

**Table 1:** Demographic and clinical characteristics of study population (*n* = 76 patients).

Demographic data	Total (*n* = 76)	Tumor 4–6 cm (*n* = 51) Group 1	Tumor > 6 cm (*n *= 25) Group 2	*P* value
Sex, *n* (%)				0.9279
Male	34 (44.7)	23 (45.1)	11 (44.0)	
Female	42 (55.3)	28 (54.9)	14 (56.0)	
Age, median(IQR)	55 (43–63)	59 (46–66)	50 (40–57)	**0**.**0418**
BMI, median(IQR)	24.3 (22.3,26.9)	24.6 (23.0,27.2)	24.0 (21.4,25.9)	0.3668
Clinical presentation, *n* (%)				0.5086^a^
Incidentaloma	36 (47.4)	22 (43.1)	14 (56.0)	
High blood pressure	8 (10.5)	6 (11.8)	2 (8.0)	
Pain	8 (10.5)	4 (7.8)	4 (16.0)	
Oncologic extension workup	7 (9.2)	6 (11.8)	1 (4.0)	
Palpitation	5 (6.6)	3 (5.9)	2 (8.0)	
Others	12 (15.79)	10 (19.61)	2 (8.00)	
Functioning features, *n* (%)				0.9482^a^
Nonfunctioning	45 (59.2)	30 (58.8)	15 (60.0)	
Aldosterone	8 (10.5)	6 (11.8)	2 (8.0)	
Cortisol	4 (5.3)	3 (5.9)	1 (4.0)	
Catecholamine	19 (25.0)	12 (23.5)	7 (28.0)	
Tumor side, *n* (%)				0.4564
Left	26 (34.2)	16 (31.4)	10 (40.0)	
Right	50 (65.8)	35 (68.6)	15 (60.0)	
Adrenal malignancy, *n* (%)				0.6809
Benign	47 (61.8)	30 (58.8)	17 (68.0)	
Malignant	20 (26.3)	14 (27.5)	6 (24.0)	
Secondary metastasis	9 (11.8)	7 (13.7)	2 (8.0)	
Operative time (mins), median (IQR)	150 (114–193)	135 (105–165)	175 (145–230)	**0**.**0053**
Estimated Blood loss (cc), median (IQR)	50 (0–163.5)	Minimal (0–100)	100 (0–300)	**0**.**0470**
Estimated Blood loss subset, *n* (%)				0.2889
<50 cc	37 (48.7)	27 (52.9)	10 (40.0)	
≥50 cc	39 (51.3)	24 (47.1)	15 (60.0)	
Open Conversion (%)				**0**.**0327**^a^
Yes	3 (4.0)	0	3 (12.0)	
No	73 (96.0)	51 (100)	22 (88.0)	
Intra-operative complications, *n* (%)				0.1058^a^
Yes	8 (10.5)	3 (5.9)	5 (20.0)	
No	68 (89.5)	48 (94.1)	20 (80.0)	
Postoperative complications, *n* (%)				1.0000^a^
Yes	5 (6.6)	3 (5.9)	2 (8.0)	
No	71 (93.4)	48 (94.1)	23 (92.0)	
Length of hospital stay, median (IQR)	5 (4–7)	5 (4–7)	5 (5–7)	0.3738

Statistical significances were noted between groups: age, operative time (mins), estimated blood loss (cc.), conversion to open approach.

^a^ Fisher's exact test.

The bold values indicate statistically significant differences (*P* < 0.05).

The median BMI value for Group 1 was 24.6, and the median BMI value for Group 2 was 24.0. No significant differences were found in BMI between these two groups.

Adrenal tumors were found incidentally in 36 patients (47.4%). Other clinical presentations included hypertension (*n* = 8, 10.5%), pain (*n* = 8, 10.5%), oncological work-up (*n* = 7, 9.2%) and palpitations (*n* = 5, 6.6%). Thirty-one (40.8%) patients presented with a functional tumor, 4 (5.3%) had cortisol secretion, 19 (25%) had catecholamines secretion, and 8 (10.5%) had aldosterone secretion.

RLA was performed on the left side in 26 patients (34.2%) and on the right side in 50 patients (65.8%). There is no significant differences were found in the clinical presentation and functioning features between these two groups (Table 1).

A total of 47 patients had benign tumors, including 30 patients in Group 1 and 17 patients in Group 2. Twenty adrenal tumors were malignant, including 14 in Group 1 and 6 in Group 2. Nine patients had metastasis from other organ malignancies, 7 in Group 1 and two in Group 2. Malignancy analysis of these tumors showed no statistically significant differences between the two groups, *P* = 0.6809.

The median operative time for Group 1 was 135 min (IQR: 105–165 min), while the median operative for Group 2 was 175 min (IQR: 145–230 min), showing a statistically significant longer operative time in Group 2 (*P* value = 0.0053). For EBL, median blood loss for all patients was 50 cc. (IQR: 0–163.5 cc). EBL in Group 1 was almost negligible (minimal amount, IQR: 0–100 cc), while the median for Group 2 was 100 cc (IQR: 0–300 cc), significantly higher than in Group 1. However, EBL analysis can be skewed by a single complex surgery with substantial blood loss, so blood loss was categorized into two groups (less than 50 cc and more than 50 cc) for comparison, showing no statistically significant differences between the two groups.

Regarding open conversion, three patients in Group 2 were converted to an open approach due to complex surgeries, significantly different from Group 1 (*P* value = 0.0327).

No significant differences were found between the two groups in intra-operative complications, postoperative complications, and length of hospital stay (*P*-values = 0.106, *P* = 1.0, and *P* = 0.37, respectively) (Table 1).

The most common intra-operative complication is hemodynamic instability, these complications are specifically associated with the treatment of pheochromocytomas, with two cases each in group 1 and group 2. Other intra-operative complications include tumor rupture during handling, with one instance in each group. Major intraoperative bleeding requiring transfusions occurred exclusively in group 2, with two cases.

Post-operative complications included pulmonary edema, with one case in each of group 1 and group 2, and post-operative low blood pressure, with one case in each group as well.

**Figure 1:**
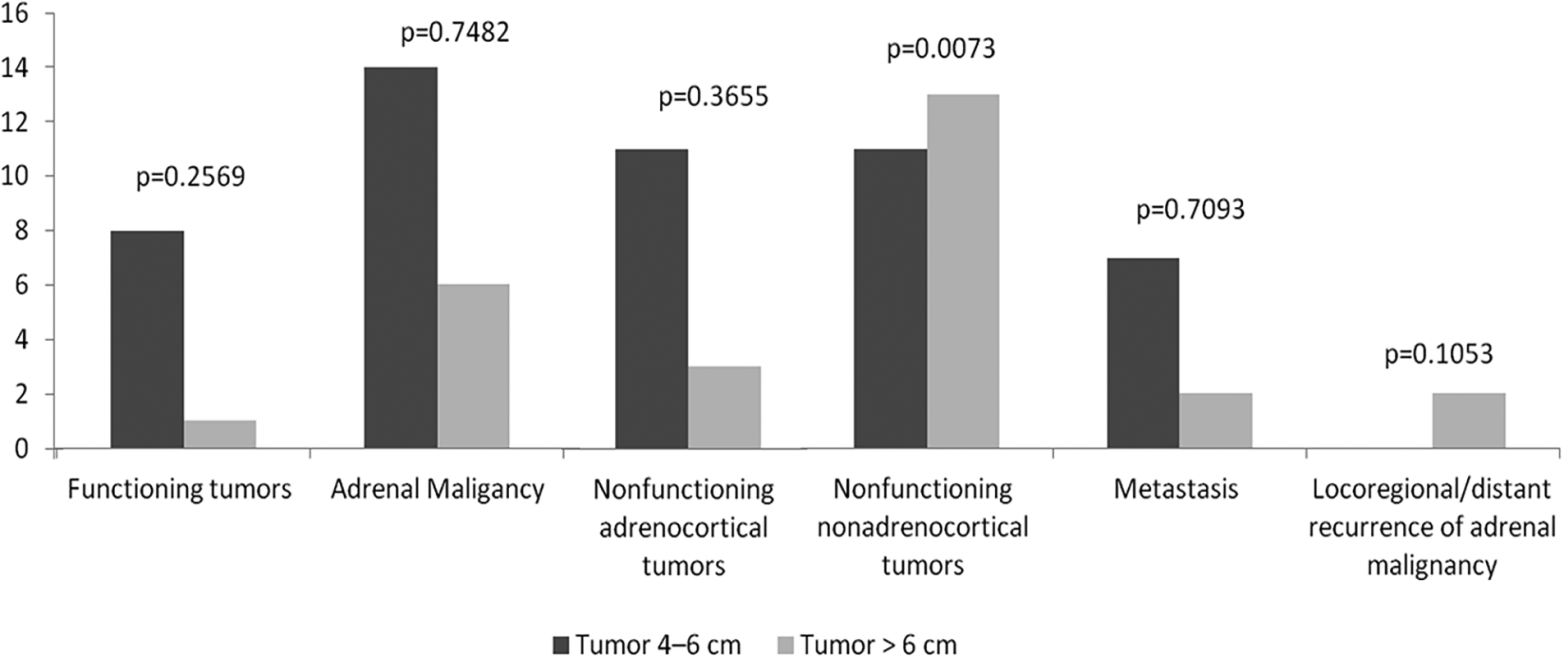
Functional and histopathologic diagnoses. No statistically significant differences were found between two groups in benign functioning tumors, adrenal malignancy and non-functioning adrenocortical tumors. Non-functioning non-adrenocortical tumors showed a statistically significant increase in patients in Group 2.

Histopathologic analysis of the tumors is shown in Table 2 and Figure 1. Benign functional tumors, including hyperaldosteronism and Cushing's syndrome, were found in both groups, 8 in Group 1 and 1 in Group 2. Adrenal origin malignant tumors, including pheochromocytoma and adrenocortical carcinoma, were also found in both groups, including 14 in Group 1 and 6 in Group 2. Nonfunctioning adrenocortical tumors (adrenal adenomas with no functionality) in both groups included 11 in Group 1 and 3 in Group 2. No statistically significant differences were found between these two groups in all of the above analyses.

**Table 2:** Functional and histopathologic diagnoses.

	Tumor 4–6 cm (*n* = 51)	Tumor > 6 cm (*n* = 25)	*P* value
Benign Functioning tumors, *n* (%)	8 (15.7)	1 (4.0)	0.2569^a^
Adrenal Malignancy, *n* (%)	14 (27.5)	6 (24.0)	0.7482
Nonfunctioning adrenocortical tumors, *n* (%)	11 (21.6)	3 (12.0)	0.3655^a^
Nonfunctioning non-adrenocortical tumors, *n* (%)	11 (21.6)	13 (52.0)	**0** **.** **0073**
Metastasis, *n* (%)	7 (13.7)	2 (8.0)	0.7093^a^
Locoregional recurrence of adrenal malignancy	0	2	0.1053^a^

Functioning tumors include hyperaldosteronism and cushing syndrome.

Adrenal Malignancy includes pheochromocytoma and adrenocortical carcinoma.

Nonfunctioning non-adrenocortical tumors include myelolipoma, ganglioneuroma, schwannoma, adrenal cyst, hemangioma and mature teratoma.

Non-functioning non-adrenocortical tumors showed a statistically significant increase in patients in Group 2.

^a^ Fisher's exact test.

The bold values indicate statistically significant differences (*P* < 0.05).

Non-functioning non-adrenocortical tumors (myelolipoma, ganglioneuroma, schwannoma, adrenal cyst, hemangioma, and mature teratoma) were found in both groups, with 11 in Group 1 and 13 in Group 2. This category showed a statistically significant increase in patients in Group 2.

In 9 patients, adrenalectomy was performed due to metastatic disease (four with metastatic hepatocellular carcinoma, three with metastatic lung cancer, one with metastatic gall bladder cancer, and one with metastatic melanoma). No statistically significant differences were found in locoregional recurrence between two groups. Two patients had tumor recurrence after tumor resection in Group 2. One was RLA performed for 8 cm pheochromocytoma, and after 53 months, recurrent 7.5 cm tumor was found, pT4N0. Another recurrence event was RLA performed for 11.5 cm adrenal cortical carcinoma, in which severe adhesion with nearby tissue and vessels was found during the surgery, therefore, the surgery was converted to an open approach, but recurrence with metastasis still occurred after 6 months.

A total of 14 surgeries experienced adverse events, including 5 patients with intraoperative complications, 2 patients with postoperative complications, 4 patients with EBL greater than 500 cc, and 3 patients required conversion to open surgery. If a surgery had multiple adverse factors, only one adverse event would be recorded per surgery. Regarding tumor size, tumor resections with adverse events had larger tumors (median size of 6 cm, IQR: 5.3–8.5 cm) (*P*-value = 0.033). Adverse events were more likely to occur in malignant adrenal tumors undergoing RLA (*n* = 8, 57.1%, *P* = 0.0018). Patients with the occurrence of adverse events also had prolonged operation times (185 min, IQR: 138–260 min, *P* = 0.0134) and prolonged hospital stays (8 days, IQR: 5–10, *P* = 0.018) as expected. No statistically significant differences were found between the two groups in sex, age, clinical presentation, functioning features, or tumor side (Table 3).

**Table 3:** Analysis of adverse events (*n* = 76 patients).

Demographic data	Total (*n* = 76)	Without adverse events (*n* = 62)	With adverse events (*n* = 14)	*P* value
Tumor size, median (IQR)	5.0 (4.4–7.0)	5.0 (4.2–6.8)	6.0 (5.3–8.5)	**0**.**0334**
Sex, *n* (%)				0.8756
Male	34 (44.7)	28 (45.2)	6 (42.9)	
Female	42 (55.3)	34 (54.8)	8 (57.1)	
Age, median (IQR)	55.5 (43.0–63.5)	54.5 (43.0–63.0)	59.5 (40.0–64.0)	0.6105
Clinical presentation, *n* (%)				0.1696^a^
Incidentaloma	36 (47.4)	32 (51.6)	4 (28.6)	
High blood pressure	8 (10.5)	5 (8.1)	3 (21.4)	
Pain	8 (10.5)	6 (9.7)	2 (14.3)	
Oncologic extension workup	7 (9.2)	5 (8.1)	2 (14.3)	
Others	12 (15.8)	11 (17.7)	1 (7.1)	
Palpitation	5 (6.6)	3 (4.8)	2 (14.3)	
Functioning features, *n* (%)				0.0698^a^
Nonfunctioning	45 (59.2)	39 (62.9)	6 (42.9)	
Aldosterone	8 (10.5)	8 (12.9)	0	
Cortisol	4 (5.3)	3 (4.8)	1 (7.1)	
Catecholamine	19 (25.0)	12 (19.4)	7 (50.0)	
Tumor side, *n* (%)				0.5367^a^
Left	26 (34.2)	20 (32.3)	6 (42.9)	
Right	50 (65.8)	42 (67.7)	8 (57.1)	
Adrenal malignancy, *n* (%)				**0**.**0018**^a^
Benign	47 (61.8)	44 (71.0)	3 (21.4)	
Malignant	20 (26.3)	12 (19.3)	8 (57.1)	
Secondary Metastasis	9 (11.8)	6 (9.7)	3 (21.4)	
Operative time (mins), median (IQR)	150 (114–193)	148 (106–175)	185 (138–260)	**0**.**0134**
Hospital stay, days, median (IQR)	5 (4–7)	5 (4–7)	8 (5–10)	**0**.**0180**

Surgeries with adverse events are statistically significantly associated with larger tumor size, longer operative time, more hospital days, and adrenal malignancy.

^a^ Fisher's exact test.

The bold values indicate statistically significant differences (*P* < 0.05).

**Figure 2:**
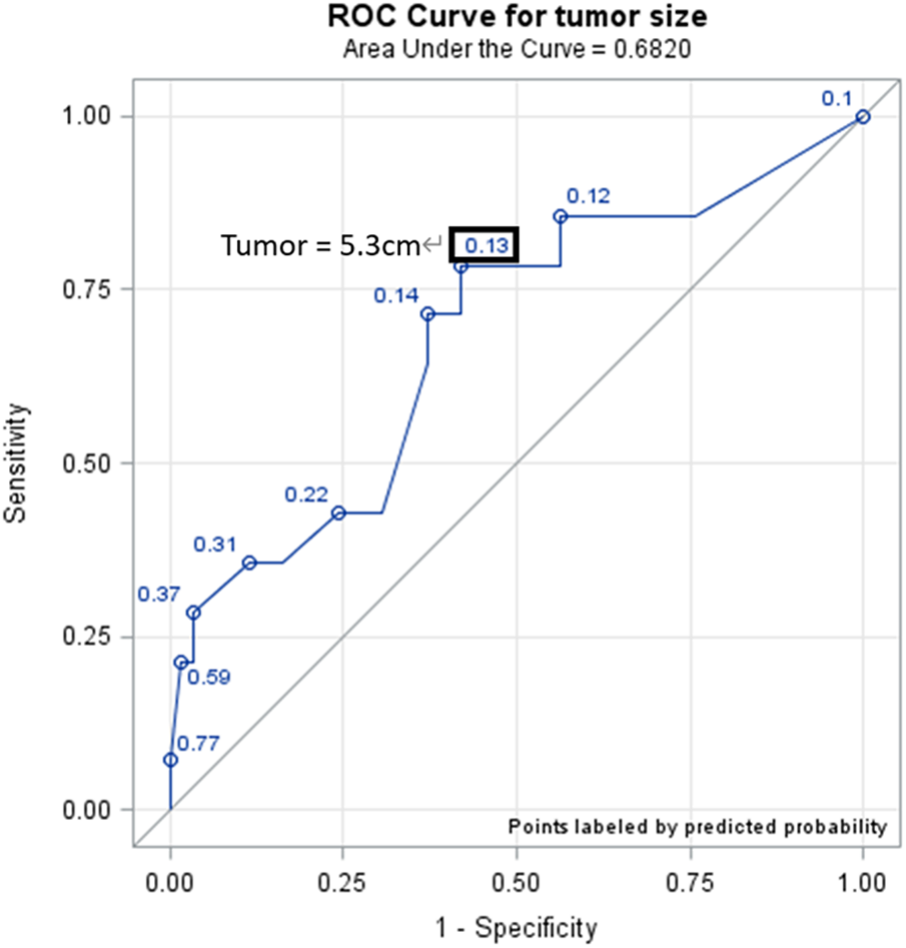
ROC curve (tumor size cutoff). The optimal cutoff point of risk of adverse events, determined by the ROC curve, is a tumor size of 5.3 cm.

In Table 4, univariable analysis for the occurrence of adverse events revealed that the unadjusted odds ratio for each additional centimeter increase in tumor size was 1.51. In multivariable analysis, the adjusted odds ratios for each additional centimeter increase in tumor size was 1.58. Statistical significance was found in both analyses, implying that for every one-centimeter increase in tumor size, there is a 1.58-fold increase in the chance of an adverse event occurring. The optimal cutoff point for tumor size regarding the occurrence of adverse events was determined to be 5.3 cm using the ROC curve (Figure 2). As seen in Table 4, the odds ratio for the occurrence of an adverse events for tumor sizes greater than 5.3 cm was 4.98, with statistical significance at *P* = 0.0492. This indicates that special attention should be given to the risk of adverse events during surgery if the tumor size exceeds 5.3 cm, the probability increases 4.98 times. Other variables, such as age, functioning features, tumor side, operative time, or adrenal malignancy, did not show a statistically significant increase in adverse events in the multivariable analysis.

**Table 4:** Univariate and multivariable analysis for occurrence of adverse events.

	Univariate analysis			Multivariable analysis^a^		
	OR	95% CI	*P* value	OR	95% CI	*P* value
Tumor size	1.51	1.09–2.09	**0**.**0122**	1.58	1.03–2.42	**0**.**0346**
Tumor size group						
≤5.3 cm	ref			ref		
>5.3 cm	3.46	0.98–12.26	0.0543	4.98	1.01–24.68	**0**.**0492**
Sex						
Male	ref			ref		
Female	1.10	0.34–3.54	0.8756	2.38	0.48–11.85	0.2890
Age group						
<55	ref			ref		
≥55	1.80	0.54–5.98	0.3375	2.48	0.42–14.52	0.3151
Clinical presentation						
No	ref			ref		
Yes	2.67	0.76–9.42	0.1277	1.40	0.28–7.06	0.6832
Functioning features						
Nonfunctioning	ref			ref		
Aldosterone	0.36	0.02–8.25	0.5207	1.01	0.05–20.94	0.9927
Cortisol	2.60	0.26–25.90	0.4142	1.02	0.06–16.31	0.9872
Catecholamine	3.65	1.04–12.75	0.0428	0.31	0.01–9.40	0.5002
Tumor side						
Left	ref			ref		
Right	0.64	0.19–2.08	0.4523	0.78	0.18–3.43	0.7439
Operative time group						
<150	ref			ref		
≥150	2.50	0.71–8.83	0.1547	1.03	0.23–4.58	0.9713
Hospital stay						
Hospital days	1.37	1.09–1.72	0.0077	1.07	0.79–1.46	0.6684
Adrenal malignancy						
Benign	ref			ref		
Malignancy	9.78	2.24–42.63	0.0024	19.21	0.54–685.59	0.1052
From other Metastasis	7.33	1.20–44.96	0.0313	3.51	0.34–36.06	0.2904

Each centimeter increase in tumor size, the odds of adverse factors increase by 1.58 times. Furthermore, special attention should be given to the risk of adverse events during surgery if the tumor size exceeds 5.3 cm, the probability increases 4.98 times.

^a^ Multivariable analysis was adjusted for age, sex, clinical presentation, functioning adrenal tumor, adrenal tumor features, operative time, hospital days, and adrenal malignancy. The AUC with 95% CI was 0.8802 (0.7864–0.9740).

The bold values indicate statistically significant differences (*P* < 0.05).

## Discussion

The present study conducted a comprehensive analysis of the outcomes of LATs treated with RLA and compared the perioperative complications and outcomes between surgeries for intermediate (4–6 cm) and large (>6 cm) tumors in two separate groups of patients. No significant differences were observed between the two groups in terms of functional and histopathologic analysis, gender distribution, functioning factors, perioperative complications, and estimated blood loss. However, patients in the large-tumor group were younger, experienced longer operative times, and had a higher rate of conversion to open surgery. While intraoperative and postoperative complications were not significantly increased in either group, larger tumors increased surgery times and were more likely to require conversion to open surgery. Nevertheless, RLA is shown to be a safe and feasible procedure for adrenal tumors larger than 6 cm.

Currently, various treatment options are applied for adrenal tumors, including open surgery, transperitoneal, retroperitoneal, and robot-assisted approaches. The mainstream treatment modalities at present are TLA and RLA. A recent systematic review, grounded in retrospective studies, discovered that many research reports indicated that RLA was superior, or at least comparable, to TLA in aspects such as operation time, EBL, pain scores, complication rates, duration of hospital stay and return to normal activity (13). The Cochrane Library has conducted a systematic review of randomized control trials (RCTs) for these two treatment methods. The current conclusion is that RLA offers advantages in terms of reduced time to oral intake, time to ambulation, and long-term morbidity. However, that systematic review of RCTs could not perform a subgroup analysis due to a lack of data on patients with LATs (tumor > 6 cm) (9).

The application of RLA for managing LATs remains a challenging and contentious topic. Lei et al. (14) and Shiraishi et al. (15) shared their experiences using TLA and RLA to treat large pheochromocytomas. Their findings demonstrated that RLA was superior to TLA, yielding reduced operation time, less EBL, quicker time to ambulation, earlier time to oral intake, and shorter hospital stays (14, 15). This aligns with the outcomes highlighted in the aforementioned systematic review (9, 13). Therefore, RLA has been shown to be a superior method for treating intermediate to large size tumors, mainly due to its ability to avoid entering the abdominal cavity. This strategy mitigates the risk of postoperative ileus or intestinal adhesions as a result of contacting the gastrointestinal tract. Furthermore, RLA circumvents vital organs such as the liver and pancreas, thus preventing the occurrence of severe complications (16). However, one disadvantage of RLA is the relatively smaller working space, which requires advanced surgical skills for managing intermediate- to large-sized tumors. Therefore, a more thorough analysis should be conducted for surgeries involving intermediate to large adrenal tumors (17).

Our guiding principle for adrenal tumors is that if a medium-to-large-sized tumor has invaded surrounding tissues or organs, we will not proceed with RLA. Instead, we opt for an open approach or TLA. This strategy helps us avoid the problem of incomplete tumor resection, which can subsequently lead to positive surgical margins. We believe that RLA is only adopted for adrenal tumors that are localized and non-invasive to surrounding tissues. If a tumor is highly suspected to be malignant but shows no invasion of surrounding tissues, we still opt for RLA to remove the tumor.

Interestingly, as seen in Table 2 and Figure 1, we noted a statistically significant association between non-functioning non-adrenocortical tumors and LATs. This may suggest that these types of tumors tend to grow larger before they are detected or present with symptoms (18, 19). However, this requires further investigation and validation in larger studies.

Our results indicated that RLA can be safely performed for adrenal tumors up to 6 cm, supporting the findings of previous studies. Based on our experience, the ideal maximum size cut-off point for RLA should be 10 cm. Tumors exceeding 10 cm are almost always accompanied by adverse events. The overall surgical outcomes of RLA were satisfactory in both groups. Operation time and EBL were statistically different between groups, with the larger tumors requiring longer operative times and leading to greater blood loss. This can be attributed to the increased complexity and difficulty associated with managing larger tumors. To mitigate the impact of extreme cases on overall statistics, we divided the data into groups (EBL > 50 or <50 cc) for analysis. However, no significant differences were found in EBL between intermediate-sized and large-sized tumors.

The open conversion was more common in the large tumor group, highlighting the increased complexity and surgical challenges associated with larger adrenal masses. Despite this, the overall complication rates did not significantly differ between the groups. This suggests that while large adrenal tumors present increased surgical challenges, these can be managed effectively with RLA without necessarily leading to increased complication rates.

Our study demonstrates the association of higher odds ratios for adverse events with increasing tumor size; however, it's crucial to clarify that our findings do not assert a direct causal relationship between tumor size and an elevated risk of adverse events. For each centimeter increase in tumor size, the odds ratio for the occurrence of an adverse events was 1.58, indicating that the probability of an adverse events increases by 58%. Furthermore, we found that the optimal cutoff point for tumor size regarding the occurrence of an adverse events is 5.3 cm. Tumors larger than this threshold had an almost five-fold increase in the likelihood of adverse events compared to smaller tumors. Therefore, although RLA is a feasible option for LATs, careful consideration should be given to the size of the tumor and the patient's overall condition. Surgeons should be prepared for potential difficulties associated with LATs, such as the potential for increased blood loss, longer operation times, and the potential for conversion to open surgery. Furthermore, a multi-disciplinary approach involving a team of experienced surgeons, anesthesiologists, and radiologists is crucial in managing these complex cases.

Currently, there are relatively few prospective studies or RCTs related to LATs. This is partly because LATs account for less than 10% of all adrenal tumors. On the other hand, many hospitals do not use RLA to treat adrenal tumors, but rather use TLA or open adrenalectomy. As a result, The Cochrane Library's 2018 systemic review was unable to perform a subgroup analysis for the LATs category. Therefore, it is hoped that high-volume hospitals will undertake substantial prospective research on RLA for LATs in the future (9).

### Limitations

One of the limitations of the present study, which is a single-center study with a retrospective design and not prospectively collected data, is the relatively small sample size. This limitation may restrict the generalizability of our findings to other populations. Additionally, all surgeries in this study were performed by three experienced surgeons, which could influence the surgical outcomes. To overcome these limitations, future studies should be prospective, involve larger patient cohorts, and include surgical teams from various centers to validate and expand upon our findings.

## Conclusion

RLA for LATs is feasible and safe, but it comes with challenges. The size of the tumor significantly impacts the surgical outcomes, with larger tumors associated with longer operation times, more significant blood loss, and a higher chance of conversion to open surgery. The risk of adverse events increases with the size of the tumor. Therefore, careful preoperative evaluation and meticulous surgical technique are crucial in managing LATs. The cutoff size of 5.3 cm may serve as a useful guide for surgeons in risk stratification and surgical planning for patients with LATs.

## Data Availability

The original contributions presented in the study are included in the article/Supplementary Material, further inquiries can be directed to the corresponding author.
